# A simple and robust approach to reducing contact resistance in organic transistors

**DOI:** 10.1038/s41467-018-07388-3

**Published:** 2018-12-03

**Authors:** Zachary A. Lamport, Katrina J. Barth, Hyunsu Lee, Eliot Gann, Sebastian Engmann, Hu Chen, Martin Guthold, Iain McCulloch, John E. Anthony, Lee J. Richter, Dean M. DeLongchamp, Oana D. Jurchescu

**Affiliations:** 1 Department of Physics and Center for Functional Materials, Wake Forest University, Winston-Salem, North Carolina 27109 USA; 2 Materials Science and Engineering Division, National Institute of Standards and Technology, Gaithersburg, Maryland 20899 USA; 3 KAUST Solar Center (KSC), King Abdullah University of Science and Technology (KAUST), Thuwal, 23955-6900 Saudi Arabia; 4 Department of Chemistry and Centre for Plastic Electronics, Imperial College London, London, SW7 2AZ UK; 5 Department of Chemistry, University of Kentucky, Lexington, Kentucky 40506 USA

## Abstract

Efficient injection of charge carriers from the contacts into the semiconductor layer is crucial for achieving high-performance organic devices. The potential drop necessary to accomplish this process yields a resistance associated with the contacts, namely the contact resistance. A large contact resistance can limit the operation of devices and even lead to inaccuracies in the extraction of the device parameters. Here, we demonstrate a simple and efficient strategy for reducing the contact resistance in organic thin-film transistors by more than an order of magnitude by creating high work function domains at the surface of the injecting electrodes to promote channels of enhanced injection. We find that the method is effective for both organic small molecule and polymer semiconductors, where we achieved a contact resistance as low as 200 Ωcm and device charge carrier mobilities as high as 20 cm^2^V^−1^s^−1^, independent of the applied gate voltage.

## Abstract (web summary)

Minimizing contact effects in organic semiconductor-based devices is a key step toward the development of a low-cost technology for next-generation electronics. Here, the authors reduce contact resistance in organic devices by engineering electrodes with high work function surface domains.

## Introduction

The promise to impact contemporary applications has sparked great interest in the study of organic electronic and optoelectronic devices. The rich chemistry of organic materials, low manufacturing cost, and compatibility with flexible and stretchable substrates provide an opportunity to incorporate electronics in non-traditional areas, such as clothing, paper, flexible and rollable displays, or bio-integrated applications^1–4^. The progress in this field has been significant, and recently developed solution-processable small molecule and polymer semiconductors have reached charge–carrier mobilities *(μ)* previously reserved for inorganic materials^5–9^. A direct consequence of enhancing the intrinsic mobility of the organic semiconductor layer is that the contributions of the contact effects to the device performance now can be significant. In organic field-effect transistors (OFETs), this issue becomes more severe as the channel dimensions are minimized, since the channel resistance decreases with shrinking channel length, while the contact resistance is independent of this variable. Additionally, the development of new materials hinges on a correct evaluation of mobility: the equations adopted from silicon-based metal-oxide semiconductor field-effect transistors (MOSFETs) for the characterization of OFET operation assume negligible contact resistance, and thus they fail when the devices are severely limited by contacts. In this case, it is impossible to access the intrinsic properties of materials and to provide meaningful feedback for material design^10–13^.

The impact of contacts was recognized by many research groups^14–23^, and recently Klauk identified it as the largest hurdle to overcome in the pursuit of high-frequency OFETs^24^. Contact resistance results from the fact that a fraction of the applied voltage is necessary to transfer the charges from the electrode surface to the semiconductor layer. The magnitude of this potential drop at the contact depends on the geometry of the device, with coplanar contacts typically exhibiting higher contact resistance than staggered structures^22^, and several intrinsic factors. The energetic mismatch between the electrode work function and the transport level of the organic semiconductor hampers the injection process; a solution to this problem is to chemically tailor the electrode surface with self-assembled monolayers (SAMs)^25,26^. Often, however, these modifications also alter the surface energy of the electrodes, therefore impacting the morphology of the films deposited on these surfaces^26–28^. Charge injection layers and contact dopants have been introduced to enhance injection by increasing the charge-carrier concentration at the electrodes^16,20,29,30^. For top-contact transistors, degradation of the semiconductor layer underneath the electrodes often occurs due to the high energy of the evaporated metal particles. Methods such as nanotransfer printing or flip-chip lamination, were successfully implemented to eliminate this effect^31,32^. Other proposed solutions to eliminate degradation include the use of organic electrode materials such as graphene, reduced graphene oxide, carbon nanotubes, or charge transfer salts^33–36^. Recently, Uemura, et al. found that contact annealing can minimize the contact resistance and eliminate the non-ideal current–voltage curves arising from gated Schottky contacts^11^. Contact resistances in the hundreds Ωcm range were obtained upon SAM treatment of the source/drain contacts^26,37,38^, and even below 100 Ωcm resulting from careful control of device structure and geometry^39–41^.

Here, we demonstrate that the contact resistance in bottom-contact OFETs can be significantly reduced by optimizing the metal deposition rate in conjunction with using a SAM treatment. This resulted in over fivefold improved field-effect mobility, compared with the best previously reported devices with identical composition and structure. The approach is effective for both small molecule and polymer OFETs, and we obtained contact resistances as low as 200 Ωcm, and field-effect mobilities of 19.2 cm^2^V^−1^s^−1^ for 2,8-difluoro-5,11-bis(triethylsilylethynyl) anthradithiophene (diF-TES ADT) and 10 cm^2^V^−1^s^−1^ for indacenodithiophene-*co*-benzothiadiazole copolymer (C_16_IDTBT), with minimal dependence on the gate voltage. This step change in mobility provides the impetus to propel the performance of organic electronic devices beyond the requirements of a range of commercial applications. To understand this drastic improvement in device performance, we performed grazing incidence X-ray diffraction (GIXD) and Near Edge X-Ray Absorption Fine Structure (NEXAFS) measurements on the organic semiconductor films to evaluate whether the modification in the contact deposition procedure results in variations in the film morphology and/or microstructure, and found no major differences in the structure of the semiconductor layer. This result suggests that the improvements in device performance originate primarily from the differences in the electrode properties. We find the metal grain size correlates negatively with the deposition rate, as confirmed by atomic force microscopy (AFM) measurements, thus creating different environments for the SAM attachment and also impacting its final structure. Evaluation of the SAM/Au surfaces using scanning Kelvin probe microscopy (SKPM) indicated that there exist local enhancement regions in the work function of the electrodes fabricated using a low deposition rate, pointing to the existence of regions with more efficient charge injection due to enhanced SAM order, a feature which is absent in the samples obtained via fast metal deposition.

## Results

### Electrical characterization of diF-TES ADT OFETs

**Fig. 1:**
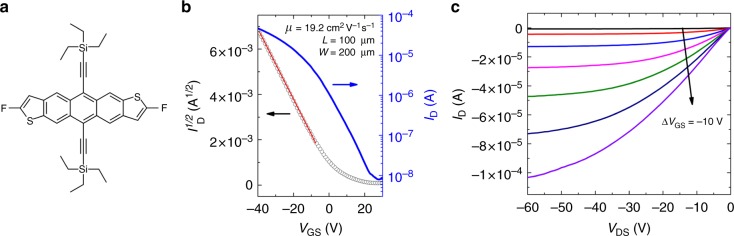
Electrical properties of OFETs fabricated on diF-TES ADT. **a** Chemical structure of diF-TES ADT. **b** Drain current as a function of gate-source voltage in device with channel length *L* = 100 µm and channel width *W* = 200 µm, in the saturation regime. **c** Drain current as a function of drain-source voltage for the same device

The chemical structure of diF-TES ADT is shown in Fig. 1a, and the electrical characteristics of a device made using a Au deposition rate of 0.5 Ås^−1^, followed by treatment with pentafluorobenzene thiol (PFBT) is depicted in Fig. 1b, c. In Fig. 1b, we show the evolution of the drain current (*I*_D_) as a function of the gate-source voltage (*V*_GS_) in the saturation regime, with the drain-source voltage (*V*_DS_) held constant at −40 V. The blue line corresponds to *I*_D_ on a log scale (right axis), and the black open circles correspond to the square root of *I*_D_ (left axis). The red line serves as a visual aid to show that the square root of *I*_D_ follows a linear relation with *V*_GS_, as expected from the gradual channel approximation, and indicates the section of the curve where the field-effect mobility was calculated. Fig. 1c shows the evolution of *I*_D_ with *V*_DS_, where each curve is measured at a different *V*_GS_, and demonstrates linearity at low *V*_DS_ and a clear transition from the linear to saturation regime. Both these features are emblematic for low contact resistances. This device exhibits a field-effect mobility of *μ*_sat_ = 19.2 cm^2^V^−1^s^−1^, a current on/off ratio of *I*_on_*/I*_off_ = 6 ∙ 10^3^, and a threshold voltage of *V*_Th_ = 3.3 V. The relatively modest on/off ratio originates from the fact that we have not patterned our device arrays. Larger *I*_on_*/I*_off_ ratios are possible, as shown for example in Supplementary Fig. 1, where *I*_on_*/I*_off_ = 10^7^. A summary of on/off ratios obtained in diF-TES ADT OFETs fabricated on source drain contacts deposited at a rate of 0.5 Ås^−1^ is displayed in Supplementary Fig. 2. The value of on/off ratio is controlled by many factors, and it is quite common that a wide spread is obtained in OFETs^38^.

In order to confirm that the mobility is not overestimated, we evaluated its dependence on *V*_GS_. Mobility overestimation can occur in the case of gated Schottky contacts, where there is a large injection barrier at the contacts, which is overcome by an increasing *V*_GS_. This relation causes a peak in the apparent mobility when the injection barrier is eliminated, before decreasing to a more realistic value^10–12^. As can be observed in Supplementary Fig. 3, the mobility in our device first increases with increasing *V*_GS_, followed by a plateau at higher *V*_GS_. Such a dependence has been observed in other high mobility systems such as C_10_DNTT thin films or rubrene single crystals and was attributed to the presence of electronic traps in the organic semiconductor layers^11,41,42^. The field-effect mobility evaluated in the linear regime for the device presented in Fig. 1 was *µ*_lin_ = 16.0 cm^2^V^−1^s^−1^ (Supplementary Fig. 4). The contact resistance has a greater effect on the effective device mobility in the linear regime, and the close correspondence recorded between the linear and saturation mobilities suggest a low contact resistance, in agreement with the linear curves obtained in the low-*V*_DS_ range of the output characteristics in Fig. 1c. A quantitative analysis of the contact resistance and its effect on device properties will be provided later.

**Fig. 2:**
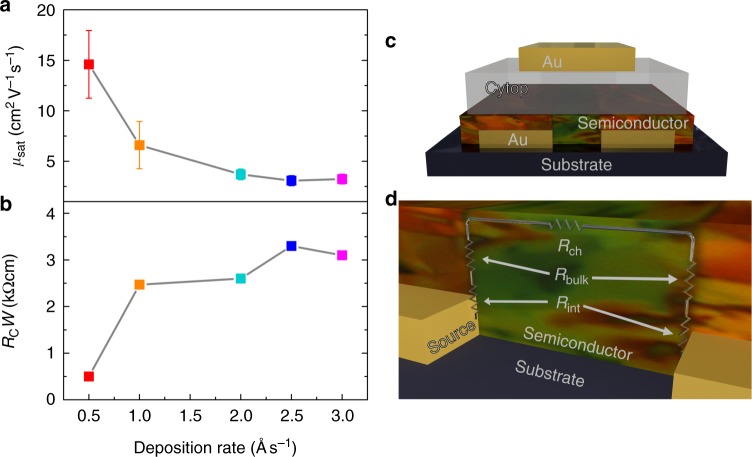
Contact resistance in OFETs. **a** Average field-effect mobility versus contact deposition rate. **b** Width-normalized contact resistance as a function of contact deposition rate. **c** Schematic of the bottom-contact, top-gate device structure used in our devices. **d** Equivalent circuit diagram including the different sources of resistance in our devices

Figure 2 shows the results of diF-TES ADT devices fabricated using varied contact deposition rates along with two device schematics for the bottom-contact, top-gate architecture. The evolution of the average field-effect mobility (i.e., the effective device mobility) with the contact deposition rate is depicted in Fig. 2a, where the error bars indicate standard deviation. This value was averaged over at least 5 devices, and the histograms for 30 devices are shown in Supplementary Fig. 5. The device architecture was identical in all samples (see Fig. 2c), and the only difference was the rate used for the deposition of the source and drain contacts. An average field-effect mobility of *µ*_sat,avg_ = 14.6 ± 3.3 cm^2^V^−1^s^−1^ was obtained when a rate of 0.5 Ås^−1^ was used, decreasing to an average field-effect mobility of *µ*_sat,avg_ = 3.24 ± 0.49 cm^2^V^−1^s^−1^ at a rate of 3.0 Ås^−1^. The lower values coincide with those reported using the same methods, materials, and device architecture, where devices fabricated with a contact deposition rate of 2 Ås^−1^ resulted in an average field-effect mobility of *µ*_sat,avg_ = 1.5 cm^2^V^−1^s^−1^ and a maximum field-effect mobility of *µ*_sat,max_ = 3.14 cm^2^V^−1^s^−1^^43^.

### Thin film microstructure

**Fig. 3:**
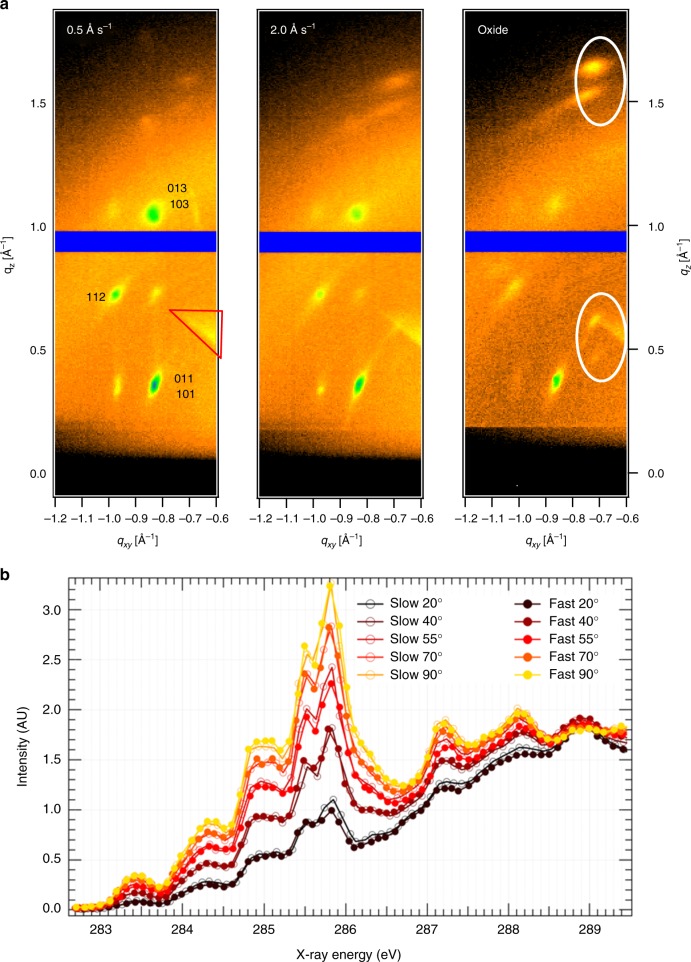
μGIWAXS and NEXAF Spectroscopy measurements. **a** μGIWAXS was recorded on the contacts of patterned device substrates. Labeled features in the left panel arise from (001) oriented crystals. The triangular feature highlighted in red, present in all images, is a background artifact. Circled features in the right panel are features from the (111) oriented crystallites present on the oxide of long channels. **b** The NEXAFS intensity of the fast and slow films at different angles between the surface normal and polarization vector of X-rays

To understand the reason behind the improvements in field-effect mobility, we first performed microbeam grazing incidence wide-angle X-ray scattering (μGIWAXS)^44^ measurements on patterned contacts of device substrates treated with PFBT and diF-TES ADT as organic semiconductor layer, but without Cytop, to assure correlation of the measurements to devices (the identification of commercial equipment or vendor is not intended to imply recommendation or endorsement by NIST, nor is it intended to imply that the materials or equipment identified are necessarily the best available for the purpose). The results for the rates of 0.5 Ås^−1^ and 2 Ås^−1^ are shown in Fig. 3a. In both cases, the mixed index peak series arising from (100) oriented crystallites can be distinguished^45^, confirming that the molecules are “edge-on” oriented, as illustrated in Supplementary Fig. 6. These findings are in agreement with earlier reports^26,45,46^. The dominant (001) orientation is a result of the PFBT-treated Au acting as a templating structure (Supplementary Fig. 7), with the fluorine atoms of the PFBT molecules interacting with the periphery of diF-TES ADT molecules^26^. This orientation is the most favorable for charge transport across the channel. Also shown in Fig. 3a is μGIWAXS from the center of long-channel devices where the PFBT templating is lost, as demonstrated by the appearance of diffraction features from the (111) crystal orientation. From Fig. 3a, it is clear that there is no significant variation in (111) fraction between the two electrodes. Additionally, there is no evidence for significant lattice strain that has been invoked as the origin of high mobility 6,13-bis(triisopropyl-silylethynyl) pentacene (TIPS-pentacene)^47^. Shown in Fig. 3b are NEXAFS results from PFBT-treated Au films deposited at 0.5 Ås^−1^ and 2 Ås^−1^, labeled as “slow” and “fast”, respectively. Partial electron-yield NEXAFS is sensitive only to the surface of the film, and thus it is most relevant to the channel of the top gate devices. The NEXAFS is remarkably similar for the different deposition rates, showing that the composition of the samples is not altered, and that the molecular orientation (predominantly “edge on” with the highest π* intensity at normal incidence) is similar at both slow and fast rates, consistent with the bulk μGIWAXS.

### Contact resistance in OFETs

Since no major differences were observed in the morphology of the film as a function of contact deposition rate, we further focused on the quantitative analysis of the changes in the contact resistance. The total device resistance *R*_device_ is given by the channel resistance, *R*_Ch_ (a quantity which is proportional to the channel length), and the contact resistance *R*_C_, as shown in Equation (1). *R*_C_ in a staggered structure, such as the ones studied here and depicted in Fig. 2c, has two main contributions: the interface resistance, *R*_int_, and the bulk resistance, *R*_bulk_, which can be seen in Fig. 2d and Equation (2). *R*_int_ is a result of the properties of the electrode surface, including the energy level mismatch between electrode and semiconductor, and the presence of interfacial dipoles, whereas *R*_bulk_ reflects the transport of the injected charges through the organic semiconductor, from the electrode/semiconductor interface to the accumulation layer. Thus, *R*_bulk_ strictly depends on the conductivity of the semiconductor in the direction perpendicular to the channel and the thickness of the semiconducting layer. The relations between the *R*_device_, *R*_Ch_, *R*_int_,, and *R*_bulk_ in the linear regime are as follows:

1 $$\begin{array}{*{20}{c}} {R_{{device}} = R_{Ch}(L) + R_{C}}\end{array}$$

2 $$\begin{array}{*{20}{c}} {R_{C} = R_{int} + R_{bulk}}\end{array}$$

We evaluated the contact resistance for the devices corresponding to each contact deposition rate using the gated transmission line method (gated TLM), based on Equation (1), and the results are displayed in Fig. 2b. The gated TLM was based on the value of *I*_D_ in the linear regime taken at an overdrive voltage of *V*_GS_ – *V*_Th_ = −40 V with *V*_Th_ determined by the second-derivative method^48^. Details about this analysis are included in Supplementary Fig. 8. It is clear from Fig. 2a, b that the increase in the average field-effect mobility as a function of contact deposition rate is mirrored by the inverse trend in contact resistance. The devices obtained using a fast deposition rate of 3 Ås^−1^ exhibit large contact resistance, *R*_C_ = 3.1 kΩcm, which yields a field-effect mobility of *µ*_sat,avg_ = 3.2 ± 0.5 cm^2^V^−1^s^−1^. By slowing down the deposition process to a rate of 0.5 Ås^−1^, we reduced the contact resistance by six times, to 500 Ωcm. Consequently, the effective mobility measured in these devices is very high. This outcome suggests, along with the identical GIXD and NEXAFS results, that the drastically improved device performance is a result of the enhanced charge injection provided by the lower deposition rates, which yields lower contact resistance. The resistance due to the distance between injection and the conduction channel, *R*_bulk_, should be unchanged between our devices fabricated using various contact deposition rates because the semiconductor has been verified to have the same crystal structure. This indicates that the main improvement in our devices lies in the resistance of the interface between the contact and the semiconductor, *R*_int_.

### Scanning probe measurements

**Fig. 4:**
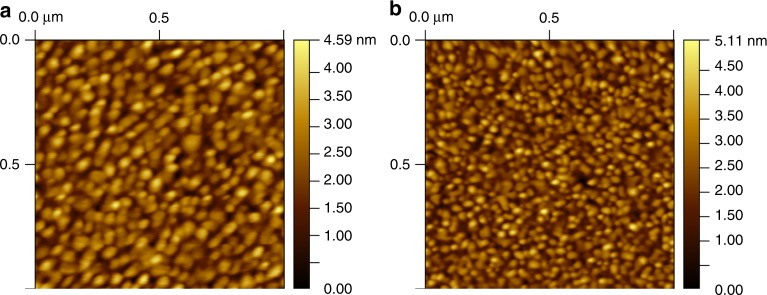
AFM images on the Au surface. **a** Au deposited at 0.5 Ås^−1^. **b** Au deposited at 2.5 Ås^−1^

Through AFM measurements, shown in Fig. 4a, b, we found that the slow deposition rate (0.5 Ås^−1^ in this case) yields a larger metal grain size than the faster rates (here 2.5 Ås^−1^), as confirmed by the 2D fast Fourier transforms in Supplementary Fig 9a, b. Au readily migrates on a substrate, a process which is enhanced by temperature. When the Au film formation is slow (similar to a simultaneous deposition and annealing), enough time is allowed for Au to reorganize to a more favorable energy state before additional particles reach the substrates, effectively “trapping” those underneath. On the contrary, at higher rates, the comparatively large amount of material reaching the surface per unit time reduces the time in which underlying Au particles can migrate from the point of initial contact to a more energetically favorable position. This process resembles that of Uemura et al., but in that case the annealing step was performed postdeposition, while here the deposition and annealing are simultaneous^11^. Nevertheless, the result is the same: lowering of the contact resistance. Interestingly, the RMS roughness of the two films are very similar (0.64 ± 0.04 nm for 0.5 Ås^−1^, 0.69 ± 0.02 nm for 2.5 Ås^−1^), suggesting that the in-plane variation is the determining factor in the field-effect mobility improvements, rather than any change in the height variation. To determine if this in-plane variation had any impact on the work function of PFBT-treated Au films, and therefore on the injection barrier, we first conducted macroscale Kelvin probe measurements and found no difference in the work function of the treated Au, both 0.5 Ås^−1^ and 2.5 Ås^−1^ gave *φ*_Au,PFBT_ = 5.3 eV. Details on the determination of work function based on Kelvin probe measurements were provided elsewhere[26].

**Fig. 5:**
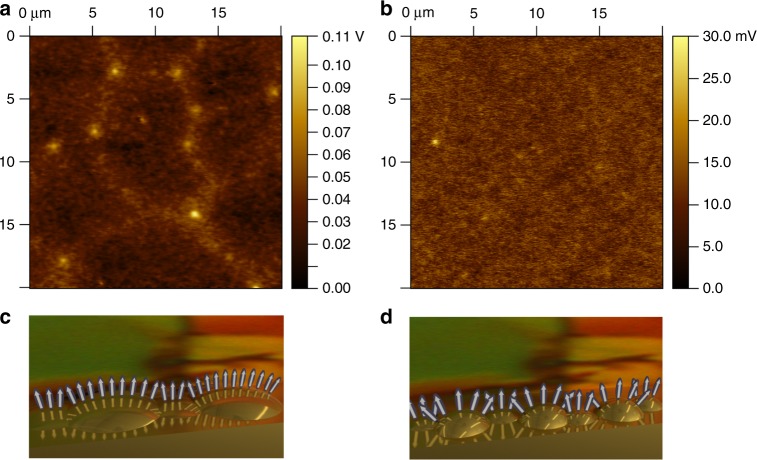
SKPM measurements on PFBT/Au. **a** PFBT-treated Au deposited at 0.5 Ås^−1^ showing small regions of higher surface potential. **b** PFBT-treated Au deposited at 2.5 Ås^−1^ showing a more homogeneous surface potential distribution. **c** An illustration of the effect of large Au grains on the assembly of PFBT, depicted here as an arrow. **d** An illustration of small Au grains on the assembly of PFBT

To examine the local features of the PFBT/Au surface potential, we performed SKPM measurements on the same samples, the results are displayed in Fig. 5. The surface potential of the PFBT-treated Au deposited at 0.5 Ås^−1^ (Fig. 5a) exhibited local peaks, a feature that does not appear in the sample obtained at a fast deposition rate (Fig. 5b). This observation indicates that while the average surface potential is very similar for all samples, variations exist on small length scales that are masked when macroscopic measurements are carried out. By combining these results with the AFM data, we conclude that the larger size of the Au grains characteristic for the films obtained at a deposition rate of 0.5 Ås^−1^ allows selected regions of the PFBT monolayer to achieve a higher degree of order (see Fig. 5c) than in the case of 2.5 Ås^−1^ deposition (Fig. 5d). Since the shift in the work function is dependent on the strength and direction of the internal dipole of the SAM, which in turn is given by the orientation and order of the SAM molecules on the surface, the local maxima correspond to a larger work function obtained in the regions where the net SAM internal dipole moment normal to the surface is maximum. The larger work function allows more efficient injection deeper into the highest occupied molecular orbital (HOMO) than the surrounding area, and thus providing lower local resistance to injection.

### Application to polymer OFETs

**Fig. 6:**
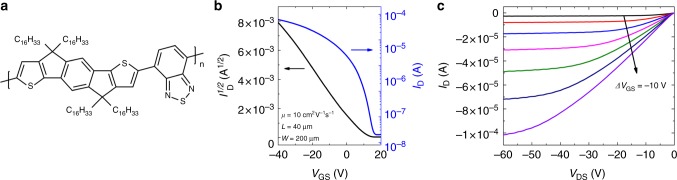
Electrical properties of C_16_IDT-BT devices. **a** Chemical structure of C_16_IDT-BT copolymer. **b** Drain current as a function of gate-source voltage for a C_16_IDT-BT device in the saturation regime (*V*_DS_ = −40V). **c** Drain current as a function of drain-source voltage for the same device

To evaluate if the enhanced hole injection from optimized contacts is effective for other semiconductors, we also evaluated FETs fabricated on polymer semiconductors. The data displayed in Supplementary Fig. 10 were obtained on the copolymer indacenodithiophene-*co*-benzothiadiazole (C_16_IDT-BT), the structure of which is displayed in Fig. 6a. C_16_IDT-BT has been incorporated as the semiconductor in many studies reaching a maximum field-effect mobility of *µ* = 3.6 cm^2^V^−1^s^−1^^49^. It can be observed that the mobility and contact resistance dependence on the contact deposition rate mirrors that obtained in small molecule devices, with the best performance resulting from a deposition rate of 0.5 Ås^−1^. Figure 6b and c show the evolution of the drain current at constant drain-source voltage while varying the gate-source voltage, and the drain current as a function of drain-source voltage with *V*_GS_ held constant, respectively. This device exhibited a field-effect mobility of 10 cm^2^V^−1^s^−1^, which is ~ 3x greater than the best mobility reported for this material in this geometry and with Cytop as dielectric^49^. Other device parameters include *I*_on_*/I*_off_ = 3 × 10^4^, *S* = 2.9 Vdec^−1^, and *V*_Th_ = 7.4 V. A histogram showing the results of 30 devices is included in Supplementary Fig. 11, and an optical micrograph of the polymer film prior to Cytop deposition is shown in Supplementary Fig. 12. These device properties are coupled with a low contact resistance of 200 Ωcm (Supplementary Fig. 13), similar to the case of the small molecule device. These results show that the reduced contact deposition rate has a strong positive effect on the device performance in polymer semiconductors as well.

## Discussion

In summary, we have enhanced the performance of our OFETs through modification of the contact deposition rate in both small molecule and polymer semiconductors. We fabricated a series of OFETs, where we varied the deposition rate for the bottom contacts between 0.5 Ås^−1^ and 3 Ås^−1^ and obtained massively improved field-effect mobility when using a rate of 0.5 Ås^−1^, reaching a value of 19.2 cm^2^V^−1^s^−1^ along with a precipitous drop in contact resistance. We conducted GIXD and NEXAFS measurements and found no noticeable difference in the microstructure of the films deposited over the substrates fabricated using the various contact deposition rates. AFM measurements confirmed a larger grain size in Au deposited at 0.5 Ås^−1^, and SKPM measurements on the same surfaces exhibited local maxima in the surface potential. We propose that these local maxima can provide regions of enhanced injection into the semiconductor, thus improving device performance, in particular for the cases when the HOMO is particularly deep compared with the work function of the electrode. Our results underline the importance of careful device fabrication in achieving high-performance organic devices. The proposed approach is efficient and robust, and it can be generally applied in all common processes and device architectures. In addition to allowing the demonstration of high-mobility transistors with near ideal current–voltage characteristics, the use of this method can also lead to accurate measurement of the charge-carrier mobility, a critical step in a rational material design, and thus providing a standardization across the field.

## Methods

### Device fabrication

Bottom-contact, top-gate devices were fabricated on both glass and SiO_2_ achieving similar results, and all data presented here are obtained on SiO_2_. The substrates were cleaned by immersion in hot acetone for 10 min, then rinsed with fresh acetone and isopropyl alcohol (IPA), followed by immersion in hot IPA for 10 min and an additional rinse using fresh IPA and dried in a stream of nitrogen. Then they were exposed to a UV–Ozone treatment for 10 min, rinsed thoroughly using deionized water and dried in a stream of nitrogen. The source and drain contacts were patterned by shadow mask and consisted of a 5 nm titanium adhesion layer deposited by e-beam evaporation at a rate of 1 Ås^−1^, followed by 40 nm of thermally evaporated gold at varying deposition rates. These contacts were then treated for 30 min using a 30 mM solution of room-temperature PFBT in ethanol followed by a 3-min sonication in fresh ethanol and a thorough ethanol rinse and dried in a stream of nitrogen. The substrates were then brought into a nitrogen glovebox (<0.1ppm O_2_, <0.1ppm H_2_O), where the organic semiconductor layer was deposited immediately. A 16.5 mg mL^−1^ solution of diF-TES ADT in chlorobenzene was spin-coated at 104 rad s^−1^ (1000 RPM) for 80 s and placed under vacuum for 90 min to remove additional solvent. C_16_IDT-BT was spin-coated at 208 rad s^−1^ (2000 RPM) for 60 s from a 10 mg mL^−1^ solution in chlorobenzene before annealing at 100 °C for 10 min. Samples were then brought back into the glovebox to apply the Cytop 809-M top-gate dielectric (*ε* = 2.1) that was spin-coated at 208 rad s^−1^ (2000 RPM) for 60 s and then annealed at 55 °C overnight resulting in a 1.4 µm film. A 40 nm gold top gate electrode was then applied using electron beam evaporation at a rate of 1 Ås^−1^.

### Device characterization

The transistor characterization measurements were carried out in the dark and under ambient conditions using an Agilent 4155 C Semiconductor Parameter Analyzer. AFM and SKPM measurements were taken using an Asylum MFP-3D Bio AFM (Asylum Research, USA) in ambient atmosphere (the identification of commercial equipment or vendor is not intended to imply recommendation or endorsement by NIST, nor is it intended to imply that the materials or equipment identified are necessarily the best available for the purpose). For AFM, a silicon cantilever (Nanosensors PPP-NCLR, force constant: 21–98 N m^−1^, resonance frequency: 146–236 kHz) was used in tapping mode with a feedback setpoint of 500 mV, and 1 µm × 1 µm images were taken at a rate of 0.5 Hz. SKPM measurements used a silicon cantilever with a Ti/Ir coating (Oxford Instruments ASYELEC.01-R2, force constant: 1.4–5.8 N m^−1^, resonance frequency: 58–97 kHz) at a nap height of 5 nm, and 20 µm × 20 µm images were taken at a rate of 1 Hz.

### μGIWAXS measurements

were performed at D-line, Cornell High Energy Synchrotron Source at Cornell University following procedures described earlier^44^. In brief, X-rays of 12 keV energy in a wide bandpass (1.47%) were focused by a single-bounce X-ray focusing capillary^50^ resulting in a nominally 15-µm transverse beam. Devices were placed on a 5-axis sample goniometer in the focal point of the capillary, with a grazing incident angle of 2° to ensure the X-ray beam projected length was less than the device electrode width. This angle of incidence exceeds the critical angle and thus the full depth of the film (and significant substrate) was probed. The devices were carefully rotated with respect to the beam footprint to enable highest spatial resolution. A Pilatus 200k image detector with a pixel size of 172 μm was placed at a distance of 180 mm from the devices. Data were reduced with the Nika software package^51^.

### NEXAFS measurements

were conducted at the Soft X-ray beam line of the Australian Synchrotron^52^, part of Australian Nuclear Science and Technology Organization, using highly linearly polarized X-rays from an elliptical polarizing undulator. Data were collected using a Channeltron detector in partial electron yield mode (retarding grid bias set at ~ 200 eV), varying the angle of the sample normal relative to the polarization vector of the incident X-rays. NEXAFS was normalized and corrected using QANT^53^.

## Electronic supplementary material

Supplementary Information

## Data Availability

The experimental data from this study are available from the corresponding author upon reasonable request.
