# Supplementary material for: A simple and robust approach to reducing contact resistance in organic transistors
Source: Nat Commun. 2018 Dec 3;9:5130. doi: 10.1038/s41467-018-07388-3 (PMC6277450; doi:10.1038/s41467-018-07388-3)
Supplement: Supplementary file 1 — Supplementary Information [file 41467_2018_7388_MOESM1_ESM.pdf]

Supplementary Information for:

A simple and robust approach to reducing contact resistance in organic transistors

Lamport, *et al.*

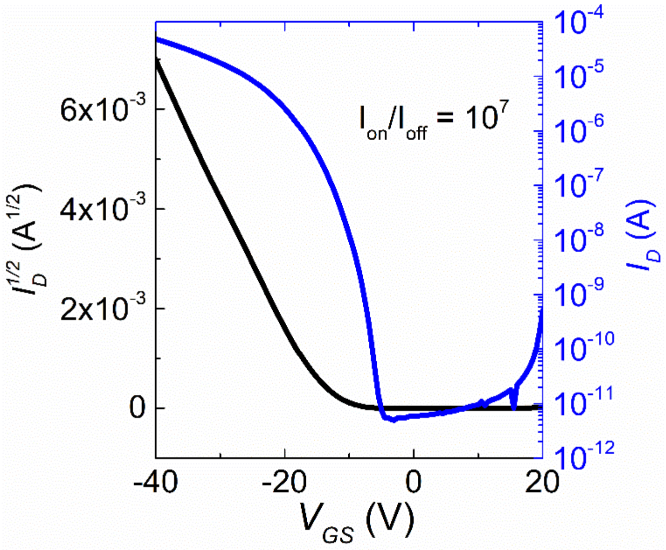

Supplementary Figure 1 | OFET characteristics for a diF-TES ADT device. Drain current as a function of gate-source voltage in a diF-TES ADT device with L = 40 μm and W = 800 μm, showing an on/off ratio greater than 10^7.

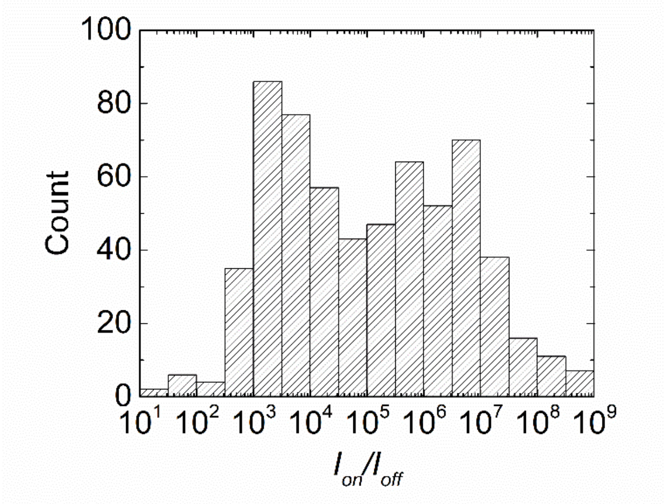

Supplementary Figure 2 | diF-TES ADT *I_{on}/I_{off}* ratios. A summary of the on/off ratios from 615 diF-TES ADT devices.

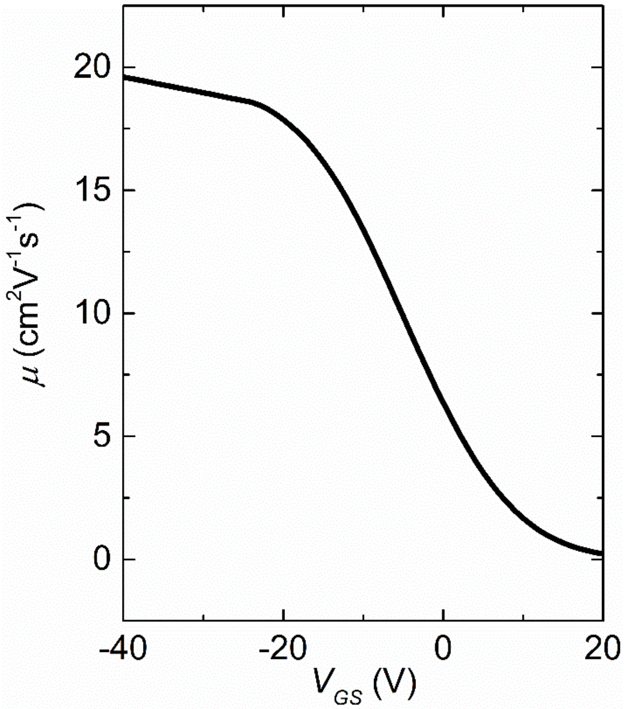

Supplementary Figure 3 | Gate voltage dependence of the field-effect mobility. Mobility vs. gate-source voltage for the device shown in Figure 1 with L = 100 µm, W = 200 µm.

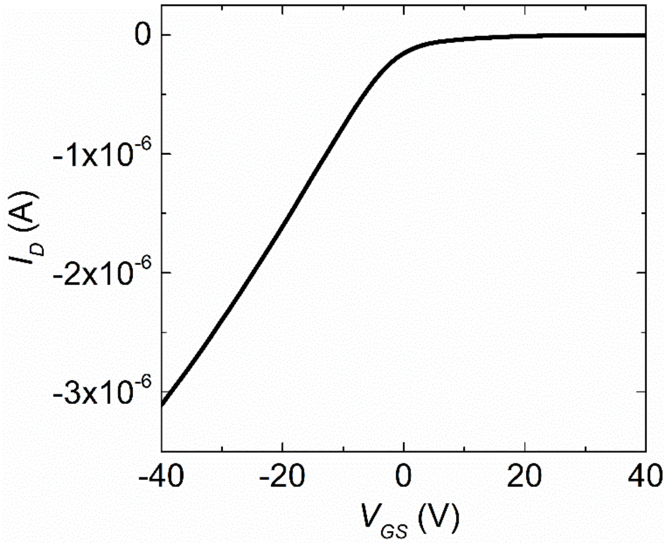

Supplementary Figure 4 | OFET characteristics of diF-TES ADT in the linear regime. I_D vs. V_{GS} in the linear regime (V_{DS} = -2V) for diF-TES ADT device for which the saturation transfer plot is shown in Figure 1b. For this device L = 100 µm, W = 200 µm, V_{Th} = 3 V, μ_{lin} = 16 cm^{2}V^{-1}s^{-1}.

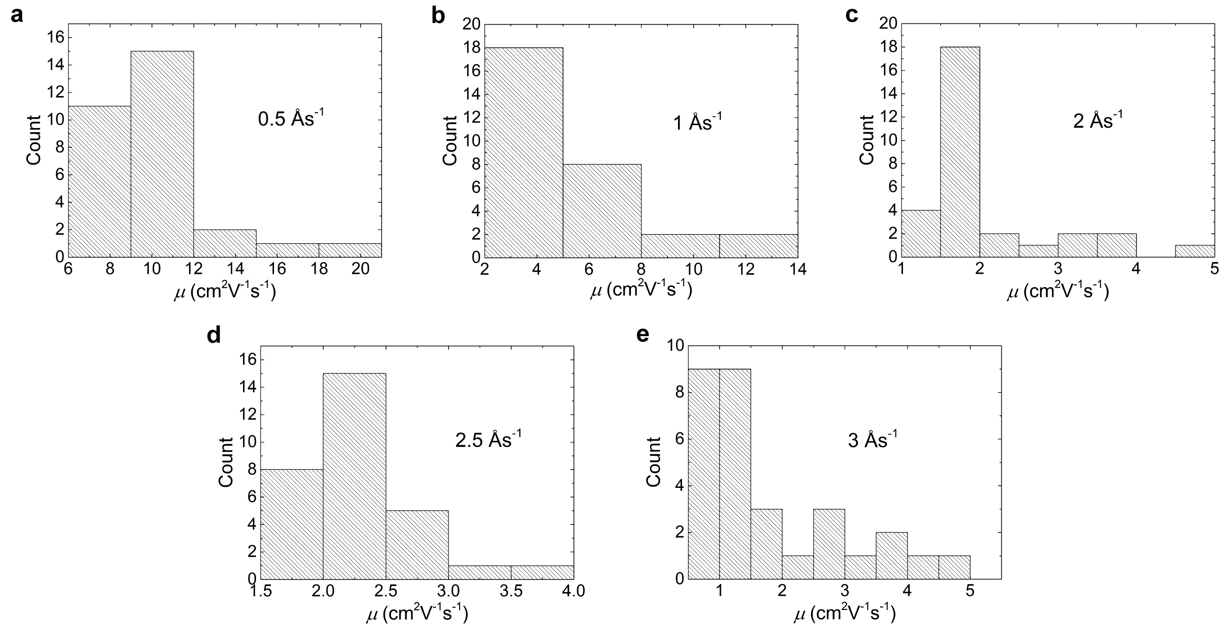

Supplementary Figure 5 | diF-TES ADT mobility statistics at varying deposition rates. The contact deposition rates are as follows: **a,** 0.5 Ås^{-1}. **b,** 1 Ås^{-1}. **c,** 2 Ås^{-1}. **d,** 2.5 Ås^{-1}. **e,** 3 Ås^{-1}.

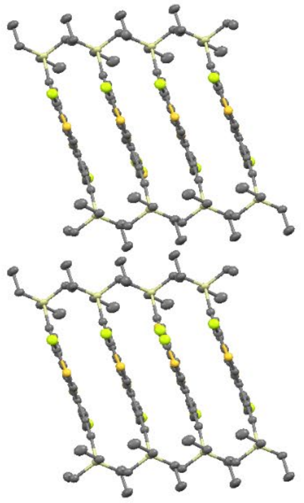

Supplementary Figure 6 | Solid state packing in the organic semiconductor. Side-view of the (001) orientation of the diF-TES ADT crystal.

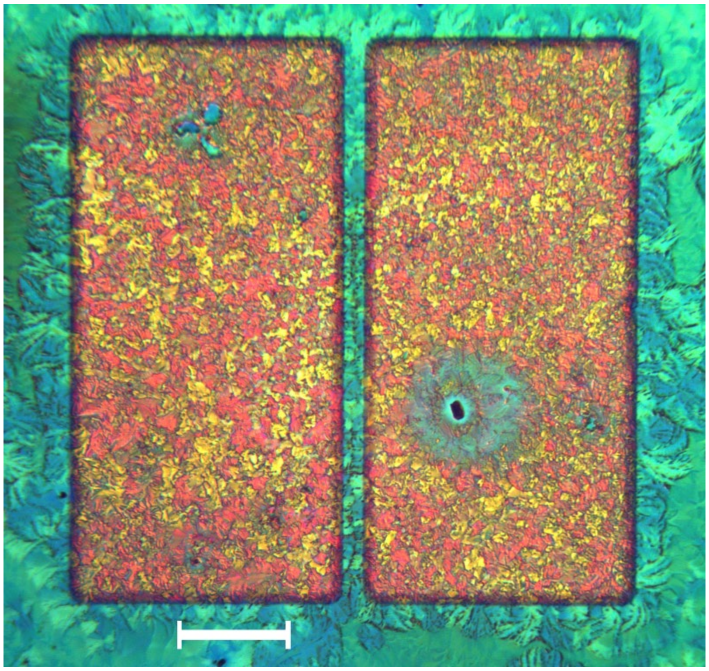

Supplementary Figure 7 | Image of a diF-TES ADT film. An optical micrograph of a spin-coated diF-TES ADT film on PFBT-treated Au showing the contact-induced crystallinity due to the interaction between the PFBT SAM and the diF-TES ADT molecules (L = 40 μm). The scale bar represents 200 μm.

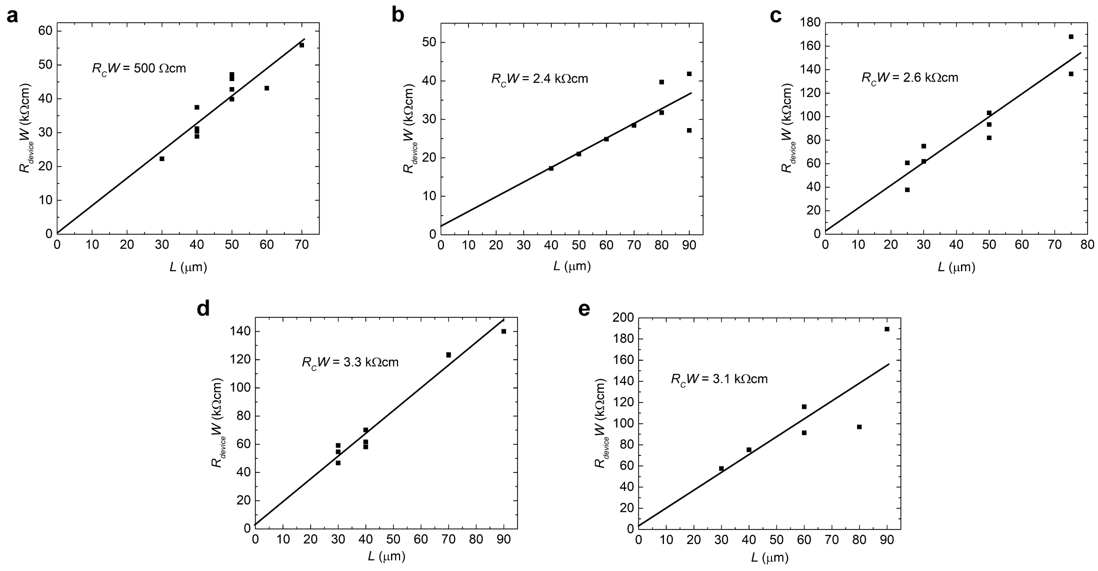

Supplementary Figure 8 | Gated transmission line method. Gated TLM measurements on diF-TES ADT OFETs fabricated with contacts deposited at varying rates. **a,** 0.5 Ås^{-1}. **b,** 1 Ås^{-1}. **c,** 2 Ås^{-1}. **d,** 2.5 Ås^{-1}. **e,** 3 Ås^{-1}.

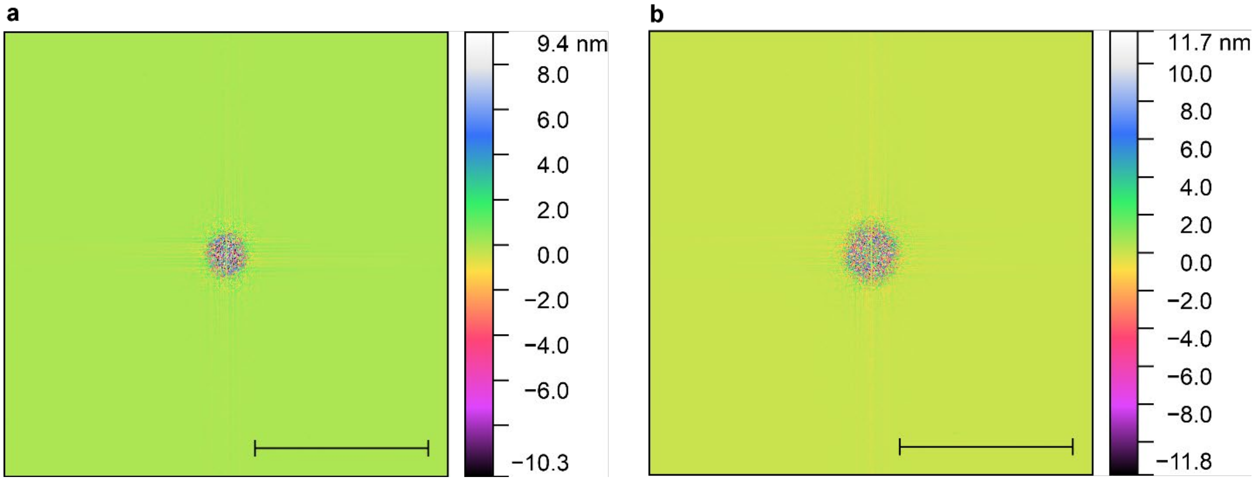

Supplementary Figure 9 | Fast Fourier transforms. 2D fast Fourier transforms to show the relative peak frequency in AFM images, and therefore the distance between metal particles, on **a**, 0.5 Ås^{-1} Au and **b**, 2.5 Ås^{-1} Au where the scale bars indicate 200 μm^{-1}. Points that are further out in reciprocal space indicate smaller spacing between particles in real space of the AFM images.

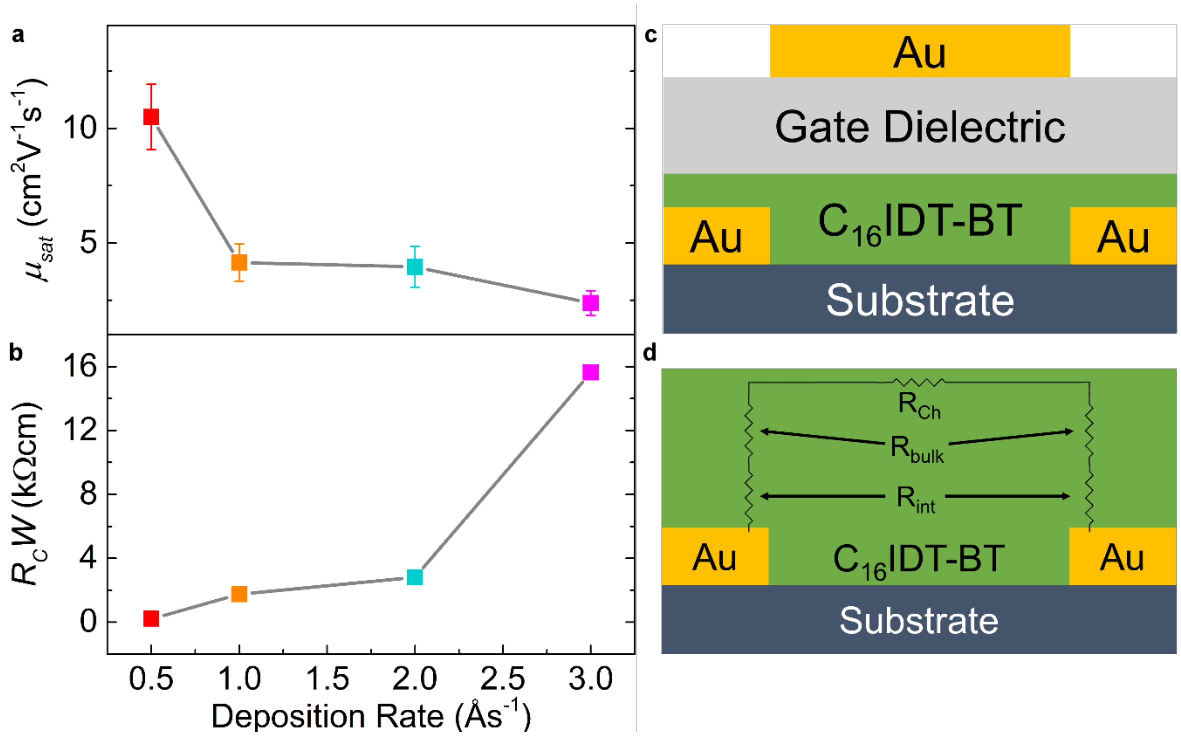

Supplementary Figure 10 | Contact resistance of C_{16}IDT-BT devices. a, Average field-effect mobility vs. contact deposition rate for C_{16}IDT-BT devices. **b,** Width-normalized contact resistance as a function of contact deposition rate. **c,** Schematic of bottom-contact, top-gate device structure. **d,** Equivalent circuit diagram showing the various sources of device resistance.

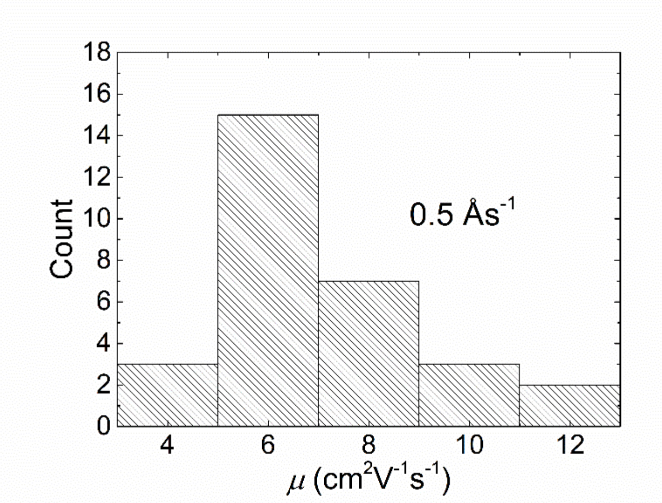

Supplementary Figure 11 | C_{16}IDT-BT devices. Histogram showing the results of 30 devices made using C_{16}IDT-BT with a contact deposition rate of 0.5 $Ås^{-1}$.

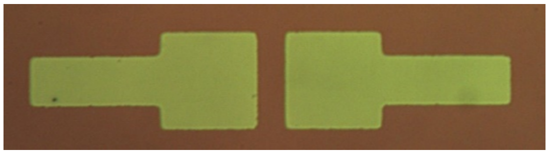

Supplementary Figure 12 | Image of a C_{16}IDT-BT film. An optical micrograph of a spin-coated C_{16}IDT-BT film on PFBT-treated Au contacts showing the uniformity of the polymer film (L = 50 μm).

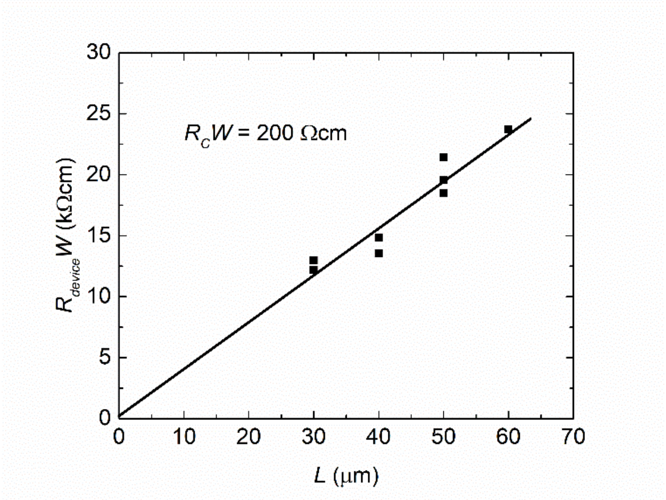

Supplementary Figure 13 | C_{16}IDT-BT contact resistance. Gated TLM measurement on C_{16}IDT-BT devices.
